# Prevalence and Characteristics of Influenza Cases From 2017 to 2019 at a Tertiary Care Teaching Hospital in Karnataka

**DOI:** 10.7759/cureus.53205

**Published:** 2024-01-29

**Authors:** Anitha Deva, Bindu Madhavi, Suresh Kumar Nagaiah, Beena PM

**Affiliations:** 1 Department of Microbiology, Sri Devaraj Urs Medical College, Sri Devaraj Urs Academy of Higher Education and Research, Kolar, IND; 2 Department of Anesthesiology and Critical Care, Sri Devaraj Urs Medical College, Sri Devaraj Urs Academy of Higher Education and Research, Kolar, IND

**Keywords:** h1n3 influenza virus, h1n2 influenza virus, h1n1 influenza virus, prevalence, severe acute respiratory illness, influenza

## Abstract

Introduction

Influenza virus is a significant human pathogen causing severe acute respiratory illness (SARI) associated with significant mortality worldwide. The H1N1 Influenza virus that caused a pandemic in 2009 continued to cause periodic epidemics worldwide, with new variants posing significant public health problems. The present study was carried out to determine the prevalence and characteristics of influenza at a tertiary care teaching hospital.

Methods

From 2017 to 2019, respiratory samples from suspected cases of influenza belonging to category C received at the microbiology laboratory were transported to Manipal Centre for Virus Research, Manipal, in the cold chain for testing of influenza virus by real-time reverse transcriptase polymerase chain reaction (rRT-PCR) as per CDC guidelines. The microbiological reports were collected and evaluated. The details of patients positive for influenza were analyzed for demographic and clinical characteristics.

Results

During the study period, 172 samples from SARI patients were tested, out of which 44 patients were positive for the influenza virus, accounting for a prevalence of 25.58%; 84% (n=37) of the cases were infected with H1N1 influenza virus, and the other 11.36% (n=5) and 4.54% (n=2) cases yielded H1N2 and H1N3 influenza virus, respectively. Among 44 patients, 56.81% (n=25) were females and 43.18% (n=19) were males. Most of the patients, 65.9% (n=29), were between 40 and 60 years old. The predominant presenting symptoms were fever in 81.81% (n=36) patients, breathlessness in 56.8% (n=25) patients, and cough in 54.54% (n=24) patients. Twelve (27.27%) patients had acute severe respiratory distress syndrome (ARDS). A significant mortality rate of 22.72% (n=10) was noted in the study.

Conclusion

A significant prevalence of influenza was noted in the study at 25.58%. Along with the H1N1 Influenza virus, the new strains detected in our region were H1N2 and H1N3 influenza viruses. Regular surveillance is important in the early detection of cases, for timely management, to reduce mortality, and to take measures to prevent the spread of this important infectious disease.

## Introduction

The influenza virus is an important human pathogen causing acute respiratory tract infections ranging from mild flu-like illness to severe acute respiratory illness (SARI) associated with significant morbidity and mortality worldwide. The rate of communicability of the influenza virus is high, leading to rapid spread from one person to another and hence can cause widespread epidemics and even pandemics [1]. Antigenic variations in influenza viruses due to genetic reassortment are responsible for their unique characteristics of rapid spread and also for the varying spectrum of clinical presentation ranging from mild flu to SARI. Thus, Influenza viruses have a high impact on the general population with regard to disease burden, morbidity, and mortality [2]. The H1N1 influenza virus that caused a pandemic in 2009 continued to cause periodic epidemics worldwide, including in India, leading to significant global public health problems. Timely diagnosis of these periodic outbreaks in different regions is important in instituting appropriate preventive and control measures in the community [3-5]. There is a paucity of data regarding the influenza virus subtypes circulating in our region. Adding on to this, there was a notable increase in the number of undiagnosed SARI cases presenting to our tertiary care hospital during 2017. Hence, the present study was carried out to determine the prevalence, subtypes, and characteristics of influenza in our region to generate knowledge on the magnitude of the disease and the locally prevalent subtypes of the influenza virus. This data is essential for public health alertness and preparedness in managing and preventing influenza cases at the regional level.

## Materials and methods

This facility-based observational study was conducted at RL Jalappa Hospital and Research Centre (RLJH and RC), Kolar, Karnataka, from January 2017 to December 2019. All the clinically suspected cases of influenza belonging to category C, as per the Ministry of Health and Family Welfare (MoHFW) guidelines on categorizing influenza cases, were included in the study [6]. The demographic and clinical details of these patients were collected and recorded. Throat swabs and nasopharyngeal swabs were collected from these cases with a dacron swab in viral transport medium (VTM) and were transported to the microbiology laboratory of Central Diagnostic Laboratory Services of RLJH and RC and were stored at the appropriate temperature till testing. As a part of a government initiative by MoHFW services, our tertiary care center was mapped under the District Surveillance Unit (DSU) of Sri Narasimha Raja (SNR) Hospital at Kolar, Karnataka, for laboratory diagnosis of influenza. DSU of Kolar was, in turn, allotted under the regional testing center for influenza virus in Karnataka, Manipal Centre for Virus Research, Manipal. Hence, the collected respiratory samples from the patients were immediately transported in a cold chain via DSU, SNR Hospital, Kolar, to Manipal Centre for Virus Research, Manipal. Testing for influenza virus was done by TaqMan™ real-time reverse transcriptase polymerase chain reaction (PCR) as per CDC guidelines and as per the manufacturer's instructions [7]. The quality control and assurance of the test procedure were carried out both by internal controls provided in the kit as well as by external controls using the pooled positive and negative samples. The results were communicated to our hospital through DSU, Kolar, on a case-to-case basis.

The Institutional Ethics Committee of Sri Devaraj Urs Medical College, Sri Devaraj Urs Academy of Higher Education and Research approval was obtained for the study (no. SDUMC/KLR/IEC/135/2019-20).

## Results

During the study period from January 2017 to December 2019, 172 patients with Category C influenza-like illness presented to RLJH and RC. On testing for influenza by real-time reverse transcriptase polymerase chain reaction (rRT-PCR), 44 patients out of 172 were positive for influenza, accounting for a prevalence of 25.58% at our tertiary care hospital.

**Figure 1:**
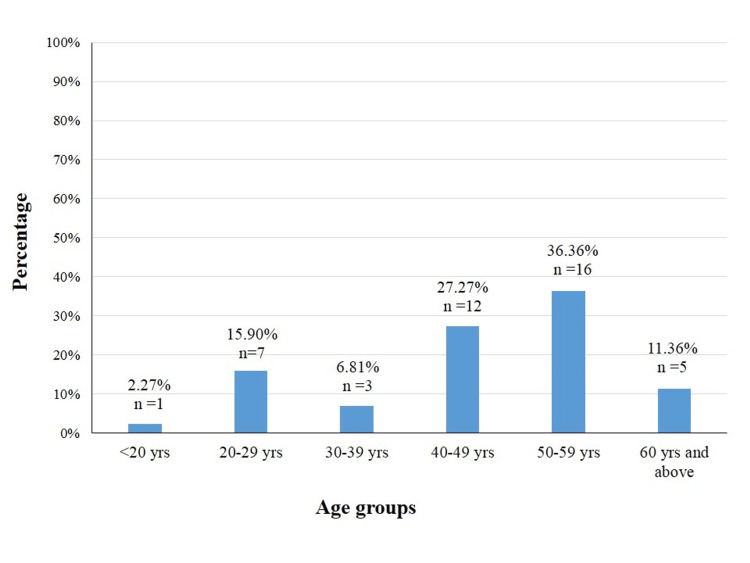
Age group distribution in influenza-positive cases

Demographic characteristics of these 44 patients showed that 56.81% (n=25) were females and 43.18% (n=19) were male patients. With regard to the age groups, the highest incidence of influenza, with 36.36% (n=16), was seen in the 50-59 years age group, followed by 27.27% (n=12) in the 40-49 years age group. Altogether, 64% (n=28) of the patients were between the age group 40-59 years, as shown in Figure 1, which is statistically significant with p-value <0.05. The lowest incidence was seen in patients aged <20 years, accounting for 2.27% (n=1). In the age groups 20-29 years, 30-39 years, and >60 years, the incidence was 15.9% (n=7), 6.81% (n=3), and 11.36% (n=5), respectively.

**Figure 2:**
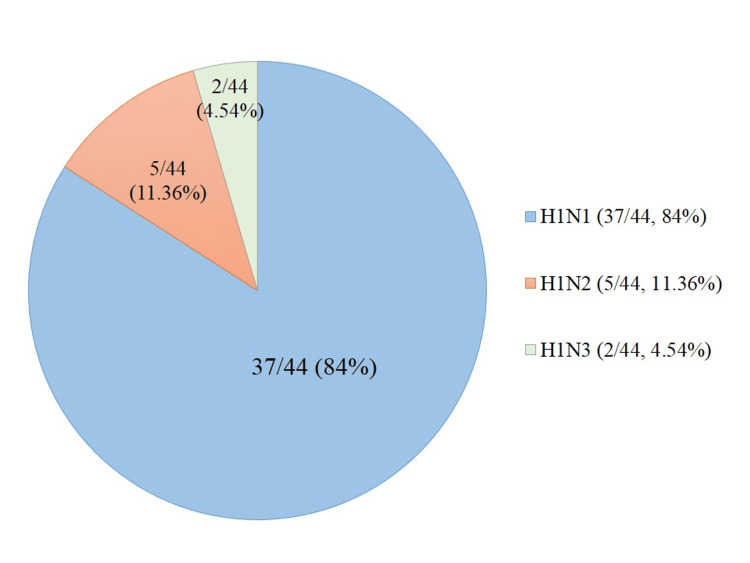
Distribution of subtypes of influenza virus

The microbiological diagnosis showed that 84% (n=37) of the cases had H1N1 influenza virus, and the other 11.36% (n=5) and 4.54% (n=2) had H1N2 and H1N3 influenza viruses, respectively, as shown in Figure 2.

The clinical characteristics of the laboratory-confirmed cases of influenza (n=44) are depicted in Table 1. Fever was present in 81.81% (n=36) of the cases, whereas 54.54% (n=24) of the cases had a cough, and 56.8% (n=25) had breathlessness. Other less common presentations were sore throat seen in 11.36% (n=5), myalgia in 13.63% (n=6), and headache in 9.09% (n=4) of the influenza cases. Associated pleural effusion was found in 9.09% (n=4) of cases. The severe form of the ARDS was noted in 27.27% (n=12) of the cases, and 20.45% (n=9) of cases required mechanical ventilation. The mortality rate among the study patients was 22.72% (n=10).

**Table 1:** Clinical characteristics of influenza cases (n=44) ARDS - acute severe respiratory distress syndrome

Clinical features	Influenza cases (n, %)
Fever	36 (81.81%)
Sore throat	5 (11.36%)
Headache	4 (9.09%)
Myalgia	6 (13.63%)
Cough	24 (54.54%)
Breathlessness	25 (56.8%)
Pleural effusion	4 (9.09%)
ARDS	12 (27.27%)
Mechanical ventilation	9 (20.45%)
Death	10 (22.72%)

## Discussion

India reported 27,236 confirmed cases and 981 deaths due to the influenza A H1N1 pdm09 strain in 2009 [3]. This novel virus continued to cause outbreaks in some parts of India and circulated in many other parts of India during the next decade [3,8-10]. During 2017-2019, our study showed a prevalence of 25.58% (44 out of 172 cases) for influenza in our region, supporting the evidence of continued circulation of this virus. Similar rates were reported by Biswas et al. in West Bengal (29.3%) [8]. During 2017 and 2018, a slightly higher prevalence was seen in other studies from Andhra Pradesh (40.45%) and Kochi, Kerala (32%) [3,11]. In contrast, slightly lower rates were reported by Chadha et al., with an overall prevalence of 14% in a multicentric study in India, and Dangi et al. reported 15.8% in Uttar Pradesh [12,13]. Factors like differences in regional temperature, humidity, surveillance categories, vaccination coverage, and different population densities and mobility might have led to variations in rates in different parts of India [14]. The study reports significant prevalence rates in our region, indicating the re-emergence of influenza. However, the cases reported in our study may represent only the tip of the iceberg, as many patients from rural areas fail to seek health care. 

Middle and older age groups were affected more in our study, which is similar to other studies [9,10]. Increased social contacts might be the possible cause for the higher involvement of adults and the elderly [15]. However, Mudhigeti et al. reported in 2018 that the most affected age group was 6-18 years (44.8%) [3]. On the other hand, Cohen et al. in 2014 found that influenza positivity was highest in the age group <5 years (49.5%) followed by 25-44 years (24.7%) [16]. In our study, 56.81 % (n=25) of the cases were females, and 43.18 % (n=19) of the cases were males, and there was no statistical significance. The other studies showed a preponderance of male patients [9,10]. 

Though H1N1 is the most common subtype found to infect the population in our region, the other influenza A subtypes detected in our study were H1N2 and H1N3, which accounted for 16% (n=7) of the cases. The findings indicate that the antigenic variations of the influenza virus continue to happen significantly, resulting in new and different strains in different geographical regions. H3N2 was another major subtype seen in Andhra Pradesh, along with H1N1 in 7.2% of the cases [3]. In a study done in Assam, among the influenza A-positive cases, 76.3% were H1N1, and 7.9% were of H3N2 subtype [9]. Influenza B was not detected in our study. Influenza B-positive cases reported in other studies belonged to both the Victoria and Yamagata lineages [3,9]. Similar findings were also reported by Heikkinen et al. in 2004 and Nandhini et al. in 2015 [17,18].

In other studies, fever was present in almost all the cases [9,15,17,18]. But in our study, only 81.81% (n=36) of the cases had fever. As the cases that were tested in our study belonged to category C as per the Ministry of Health and Family Welfare (MoHFW) guidelines on the categorization of influenza cases, cough, and breathlessness were the significant clinical features found in most of the cases, and only a few patients had a sore throat, headache, and myalgia. Similar findings were noted in other studies [3,11]. The severe form of acute respiratory distress syndrome (ARDS) was seen in 27.27% (n=12) of the patients, and most of them presented with bilateral lung infiltrates at the time of admission. These patients required mechanical ventilation. Other studies have also reported a higher incidence of mechanical ventilation in patients with initial presentation of bilateral lung infiltrates [11]. Though the mortality rate has declined over a period of time after the 2009 pandemic, few studies in India have reported a higher mortality rate [19]. Statistics from different parts of India report that the mortality rates vary from 4% to 14.8% [11,20]. However, the mortality rate in our study was quite significant, with 22.72% (n=10), which shows the severity of the illness in our region.

In 2020, the shift of diagnosis towards COVID-19 probably led to the underdiagnosis of influenza cases. There were a significant number of COVID-19 negative SARI cases in our region in 2020 and later, which were undiagnosed. Hence, the regular regional surveillance for influenza should be reconsidered so that effective preventive and control measures can be instituted on time.

The prevalence rate in our study is based on the cases presented to our tertiary care hospital alone. Even though we presume that it could indicate the prevalence in our region since our hospital is the only tertiary care hospital in this region, it may not depict the true prevalence. In addition, the cases included in the study belonged to category C influenza as per MoHFW guidelines, so the prevalence rate is of only category C influenza. The categories of A and B influenza cases were not included as they were not tested for influenza per MoHFW guidelines. These are the limitations of our study.

## Conclusions

The study brings out the local epidemiological, virological, and clinical characteristics of influenza viruses across different age groups and genders. The prevalence of 25.58% describes the significant magnitude of influenza infection in our region. Although the predominant strain was the H1N1 influenza virus, the two other strains, H1N2 and H1N3 influenza viruses, were seen in 16% of the cases is a notable finding. Regular surveillance is essential for early diagnosis and timely treatment with oseltamivir to reduce mortality. There is a need for strengthening the year-round influenza surveillance for outbreak and epidemic preparedness based on local prevalence and further for adopting influenza vaccination policies.
